# Supplementary material for: The functional evolution of termite gut microbiota
Source: Microbiome. 2022 May 27;10:78. doi: 10.1186/s40168-022-01258-3 (PMC9137090; doi:10.1186/s40168-022-01258-3)
Supplement: Supplementary file 3 — Additional file 2: Figure S1. Time-calibrated phylogenetic tree of termites inferred from mitochondrial genome sequences. Figure S2. Relative abundance of archaeal and bacterial phyla inferred from the termite gut metagenomes and the 16S rRNA amplicon data of 74 termite samples. Figure S3. Maximum likelihood phylogenetic tree inferred from 43 single-copy marker genes of 654 metagenome-assembled genomes (MAGs). The completeness and contamination of MAGs was inferred with CheckM [103]. Detailed information about each MAG is available in Table S9. Figure S4. Protein sequence alignment of predicted uricases from 53 termite transcriptomes previously published in Bucek et al. [27]. [file 40168_2022_1258_MOESM2_ESM.docx]

**Figure S1**. Time-calibrated phylogenetic tree of termites inferred from mitochondrial genome sequences.

**Figure S2**. Relative abundance of archaeal and bacterial phyla inferred from the termite gut metagenomes and the 16S rRNA amplicon data of 74 termite samples.

**Figure S3**. Maximum likelihood phylogenetic tree inferred from 43 single-copy marker genes of 654 metagenome-assembled genomes (MAGs). The completeness and contamination of MAGs was inferred with CheckM (Park *et al.*, 2015). Detailed information about each MAG is available in Table S9.

**Figure S4**. Protein sequence alignment of predicted uricases from 53 termite transcriptomes previously published in Buček et al. (2019).
